# Combination of Constraint-Induced Movement Therapy with Electroacupuncture Improves Functional Recovery following Neonatal Hypoxic-Ischemic Brain Injury in Rats

**DOI:** 10.1155/2018/8638294

**Published:** 2018-02-07

**Authors:** Hyunha Kim, Young Soo Koo, Myung Jun Shin, Soo-Yeon Kim, Yong Beom Shin, Byung Tae Choi, Young Ju Yun, Seo-Yeon Lee, Hwa Kyoung Shin

**Affiliations:** ^1^Department of Korean Medical Science, School of Korean Medicine, Pusan National University, Yangsan, Gyeongnam 50612, Republic of Korea; ^2^Korean Medical Science Research Center for Healthy Aging, Pusan National University, Yangsan, Gyeongnam 50612, Republic of Korea; ^3^Graduate Training Program of Korean Medicine for Healthy Aging, Pusan National University, Yangsan, Gyeongnam 50612, Republic of Korea; ^4^Department of Rehabilitation Medicine, School of Medicine, Pusan National University, Yangsan, Gyeongnam 50612, Republic of Korea; ^5^Division of Meridian and Structural Medicine, School of Korean Medicine, Pusan National University, Yangsan, Gyeongnam 50612, Republic of Korea; ^6^Department of Integrative Medicine, School of Korean Medicine, Pusan National University, Yangsan, Gyeongnam 50612, Republic of Korea

## Abstract

**Aim:**

Neonatal hypoxic-ischemia (HI) due to insufficient oxygen supply and blood flow during the prenatal and postnatal periods can cause cerebral palsy, a serious developmental condition. The purpose of this study was to investigate the efficacy of combining constraint-induced movement therapy (CIMT) and electroacupuncture to treat rat neonatal HI brain injury.

**Methods:**

The left common carotid arteries of postnatal day 7 rats were ligated to induce HI brain injury, and the neonates were kept in a hypoxia chamber containing 8% oxygen for 2 hrs. Electroacupuncture at Baihui (GV 20) and Zusanli (ST 36) was performed concurrently with CIMT 3 weeks after HI induction for 4 weeks.

**Results:**

Motor asymmetry after HI was significantly improved in the CIMT and electroacupuncture combination group, but HI lesion size was not improved. The combination of CIMT and electroacupuncture after HI injury increases NeuN and decreases GFAP levels in the cerebral cortex, suggesting that this combination treatment inversely regulates neurons and astrocytes. In addition, the combination treatment group reduced the level of cleaved caspase-3, a crucial mediator of apoptosis, in the cortex.

**Conclusions:**

Our findings indicate that a combination of CIMT and electroacupuncture is an effective method to treat hemiplegia due to neonatal HI brain injury.

## 1. Introduction

Neonatal hypoxic-ischemia (HI) occurs when oxygen supply and blood flow are not stable during the prenatal and postnatal periods, and this condition causes the most common form of cerebral palsy [1, 2]. Neonatal HI can cause death or brain damage leading to loss of motor ability, sensory function, and cognitive function throughout life [1, 2]. HI injury is characterized by sequential occurrences, with damage in the early phase involving cytotoxic edema, acute lysis, and oxidative stress. Later phases of HI response include inflammatory activity, apoptosis, necrosis, and long-term neural tissue repairing or remodeling [3, 4]. It was reported that 10 to 50% of patients who survived neonatal HI usually lost motor function, and 20 to 50% have lost sensory function or cognitive function [2, 5]. Despite the high incidence of neonatal HI, there is still no effective treatment for this form of injury.

In general, hemiplegia after HI has been treated by various methods, such as physical therapy, surgical treatment, and pharmacological treatment. These methods help to prevent muscular rigidity and to overcome impairment by training the patient to make skilled movements [6, 7]. Constraint-induced movement therapy (CIMT), one of the available physical therapies for rehabilitation, can induce functional recovery from hemiplegia caused by CNS injury [8]. CIMT inhibits the use of the normal arm and thus improves functional movement by encouraging the use of the damaged arm [9]. CIMT has been reported to induce neurological changes through neuroplasticity reorganization in adult HI patients, children HI patients, and animal models [10–12]. Further, it has been reported to improve motor function of the affected forelimb [13, 14]. Various modifications have been made for children undergoing CIMT because children can lack motivation and require special attention [15, 16]. Further, the efficacy of CIMT in children has been shown to be related to the type and severity of the disability [8, 17].

Electroacupuncture (EA) is a procedure that involves applying needles to acupuncture points and applying electrical stimulation. EA has been shown to be an effective treatment in stroke rehabilitation and hemiplegia [18]. Further, it has also been shown to improve not only behavioral but also neurological damage [19–21]. EA promotes neuroprotection and functional recovery in HI injury [20, 22]. In addition, previous animal studies have suggested that the therapeutic effects of EA are more pronounced when it is combined with exercise therapy [23]. The combined treatment of EA at Baihui (GV 20) and Zusanli (ST 36) along with administration of polysaccharide of Gastrodia Elata Blume was shown to increase the expression of growth factors and stem cell factor in ischemic lesions, suggesting that this combination may have the potential to repair neurological function [24].

We recently reported that CIMT yielded a modest recovery of motor function in a rat model of neonatal HI [25]. In the current study, we aimed to determine whether a combination of CIMT and EA is effective in treating HI in neonatal rats. We thus investigated behavioral improvements as well as histological changes following a combination of CIMT and EA.

## 2. Materials and Methods

### 2.1. Animals

**Figure 1:**
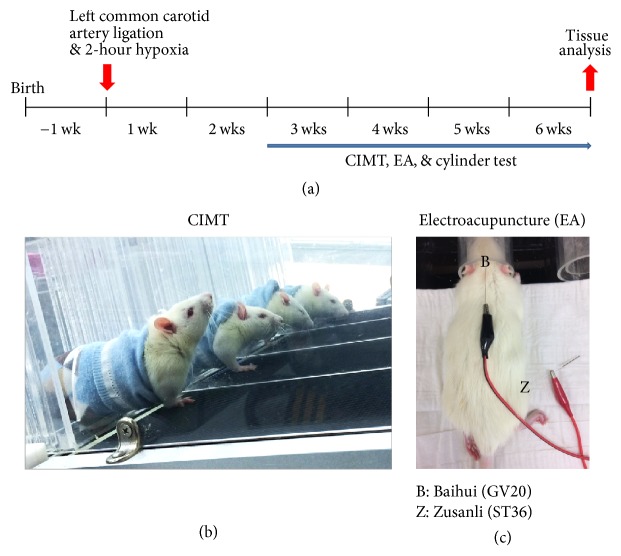
*Experimental timeline of combined CIMT and EA.* (a) Post-HI injury rat pups (7 days old) were treated with CIMT alone, EA alone, or a combination of CIMT and EA for 4 weeks. (b) To perform CIMT, the unaffected forelimb was restrained by a pouch, and use of the affected forelimb was forced by a motorized treadmill. CIMT was conducted at 3 weeks after HI injury, 10 min per day, 4 days per week, for 4 weeks. (c) EA was administered at acupoints Baihui (GV 20) and Zusanli (ST 36) 20 min per day, 4 days per week, for 4 weeks.

On day 17 (E17) of pregnancy, Sprague-Dawley rats (Doo Yeol Biotech, Seoul, Korea) were housed at 22°C under alternating 12-hour light/dark cycles. Water and food were provided ad libitum. All animal experiments followed the institutional guidelines of the Pusan National University-Institutional Animal Care and Use Committee on ethical procedures and scientific care, and all experiments were approved in advance by the institutional review board of Pusan National University (Permit number: PNU-2015-0939). Rat pups were assigned into groups using computer-generated randomization. This computer-generated randomization method assigned animals into either a sham group (*n* = 10) or an HI injury group (*n* = 36). Two weeks after inducing HI, the HI rats were randomly assigned into four groups: (1) HI injury group with no treatment (HI: *n* = 9), (2) HI injury and CIMT (CIMT: *n* = 9), (3) HI injury and EA treatment (EA: *n* = 9), and (4) HI injury and combined CIMT and EA treatment (CIMT + EA: *n* = 9).  Figure 1 shows the timeline of the animal experiments.

### 2.2. Hypoxic-Ischemic Injury Model

To induce HI injury, 7-day-old rats underwent a left common carotid artery ligation followed by exposure to 8% O_2_ for 2 hours, as previously described [25, 26]. We used both sexes because it has been reported that sex does not affect the appearance of reflexes or the extent of HI-related brain injury [27]. In brief, we ligated the left common carotid artery under general respiratory anesthesia (2% isoflurane, 80% N_2_O, 20% O_2_). The surgical temperature was maintained at 36.5–37.5°C using a Panlab temperature controlled heating mat (Harvard Apparatus, Holliston, MA). The rats were then placed in a hypoxic chamber for 2 hours. The chamber was maintained at 37 ± 0.5°C and contained an 8% O_2_/N_2_ mixture. Sham animals were anesthetized and incised but did not undergo arterial occlusion or induced hypoxia. After inducing HI injury, rat pups were returned to their mother for recovery and growth for 3 weeks. Body weight was measured 3 weeks after HI injury and monitored for 6 weeks after injury.

### 2.3. Constraint-Induced Movement Therapy

The unaffected forelimb in the CIMT group was constrained using a pouch, as previously described [25]. The rats were forced to use the affected forelimb on a motorized treadmill with a slope of 15° and a speed of 10 m/min (Figure 1(b)). CIMT began 3 weeks after HI injury and was conducted for 10 minutes per day, 4 days per week, for 4 weeks.

### 2.4. Electroacupuncture

EA treatment was also initiated 3 weeks after HI injury. The acupoints at Baihui (GV 20) and Zusanli (ST 36) were electrically stimulated (Figure 1(c)) with 2 Hz, 1 mA for 20 minutes per day, 4 days per week, for 4 weeks. Baihui (GV 20) is located at the midpoint of the parietal bone, based on the eyes and ears. Zusanli (ST 36) is located about 5 mm below the knee joint and is located at the anterior part between the tibia and the fibula [28, 29]. Electrical stimulation was conducted using a Pulse master Multichannel Stimulator SYS-A300 electrical stimulator (Word Precision Instruments, Berlin, Germany).

### 2.5. Cylinder Test

Cylinder test, also known as the spontaneous forelimb use asymmetry test, was performed to evaluate the spontaneous use of forelimb by recording the number of forelimb contacts during exploring the Plexiglas cylinder [30]. Rats were placed in a transparent Plexiglas cylinder (20 cm diameter, 30 cm height). The test is terminated when a total of 20 spontaneous forelimb-contacts (20 paws) are in the wall without time restriction. For evaluation, the preference of the rat's initial forepaw (left/right/both) was scored during rearing behavior. The forelimb asymmetry score was calculated as follows: (left − right)/total of 20 contacts.

### 2.6. Analysis of Brain Atrophy

All rats were anesthetized with isoflurane and perfused with cold PBS followed by fixation with 4% paraformaldehyde. The brain of each rat was extracted, and an image was immediately taken of the whole brain. Nissl staining was performed to measure the volumes of atrophy in each region of the brain. Briefly, the isolated brains were fixed with 4% paraformaldehyde at 4°C for 24 hours and were then dehydrated with 30% glucose for 72 hours. All brains were frozen in an optical cutting temperature medium (Sakura Finetek, Torrance, CA) and stored at −80°C until examination. Frozen brains were cut into 20 *μ*m sections using a CM 3050 cryostat (Leica Microsystems, Wetzlar, Germany). The tissue slices were stored in a storage buffer (30% ethylene glycol and 0.01% polyvinylpyrrolidone phosphate-pH 7.4) at −20°C until analysis. Each area of the brain (cortex, striatum, hippocampus, and midbrain) was measured using iSolution full image analysis software (Image & Microscope Technology, Vancouver, Canada).

### 2.7. Immunohistochemistry

All sections (20 *μ*m) were stored in a storage buffer (30% ethylene glycol and 0.01% polyvinylpyrrolidone phosphate-pH 7.4) and were permeabilized for 30 minutes with PBS containing 0.3% Triton X-100. The sections were then incubated in a blocking solution of PBS-T (0.1% tween-20 in PBS) with 10% normal goat serum and were then exposed to the following specific primary antibodies: NeuN (1 : 200, Abcam ab177487, United Kingdom), GFAP (1 : 200, Millipore MAB360, Billerica, MA), Iba-1 (1 : 200, Wako 019–19741, Japan), and cleaved caspase-3 (1 : 200, cell signaling technology 9661L, Beverly, MA). Sections were then incubated with Alexa fluor 488- (1 : 500, Thermo fisher A-11001 and A-11008, Waltham, MA) or Alexa fluor 594-conjugated secondary antibodies (1 : 500, Thermo fisher A-11005 and A-11037) for 2 hours in the dark. We focused on the slices locating at 1,21 and −3.60 mm from the bregma and dashed red square in the cortex indicates the photographed area (Supplementary Figure S1). The fluorescence images were captured with a Zeiss LSM 700 laser scanning confocal device (Carl Zeiss, Jena, Germany), and image analysis was performed using ImageJ software (NIH, Bethesda, MD).

### 2.8. Statistical Analysis

Data are expressed as mean ± standard error (SEM). The differences between the sham and HI groups were evaluated using unpaired *t*-tests. Comparison between the HI and treatment groups was made using repeated measurement one- or two-way ANOVAs followed by Tukey's post hoc tests. A *P* value of less than 0.05 was considered statistically significant. All statistical analyses were performed using SigmaPlot 11.2 (Systat Software Inc., San Jose, CA).

## 3. Results

### 3.1. Locomotor Asymmetry Is Improved by the Combination of CIMT and EA

**Figure 2:**
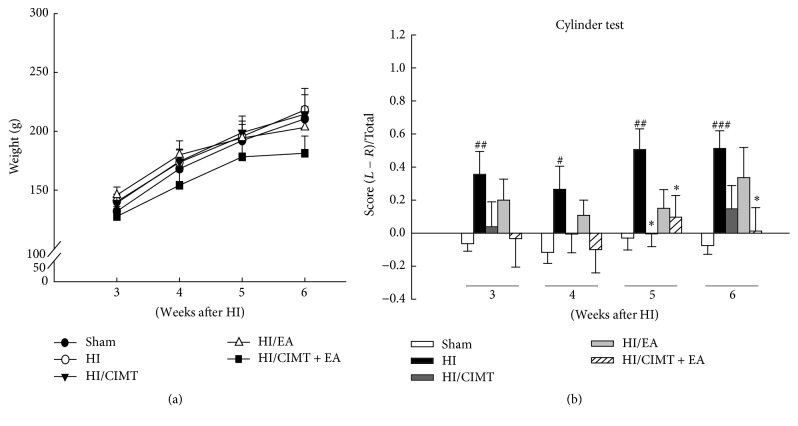
*Effects of CIMT and EA on body weight and cylinder test*. Five groups were examined. (1) No HI injury or treatment (Sham: *n* = 10), (2) HI injury (HI: *n* = 9), (3) HI injury and CIMT (HI/CIMT: *n* = 9), (4) HI injury and EA (HI/EA: *n* = 9), and (5) HI injury and combined CIMT and EA (HI/CIMT + EA: *n* = 9). (a) Body weight was observed for 3 weeks after induced HI injury. (b) The use of the preferred forelimb during rearing for the cylinder test. ^#^ *P* < 0.05, ^##^ *P* < 0.01, and ^###^ *P* < 0.001 versus the sham group (unpaired *t*-test). ^*∗*^ *P* < 0.05 versus the HI group (two-way repeated measures ANOVA).

Two pups died 3 weeks after HI injury, and another 2 pups died during the CIMT and EA treatment period. Survival rate was thus 94.44% at 3 weeks and 94.11% at 6 weeks after HI (data not shown). Thirty-two pups underwent CIMT, EA, or a combination of the two for 4 weeks after HI injury (Figure 1). Body weight was similar in all groups prior to treatment and did not significantly decrease in any of the groups throughout the period of measurement (Figure 2(a)).

Rehabilitation of locomotor asymmetry after HI was investigated using the cylinder test (Figure 2(b)). The HI group preferred the use of the unaffected forelimb compared to the sham group. Use of the affected forelimb in the HI/CIMT group was significantly increased at 5 weeks after HI, while use of the affected forelimb in the HI/CIMT + EA group was significantly increased at both 5 and 6 weeks after HI. These results suggest that the combined treatment of CIMT and EA increases the use of the affected limb and thus improves forelimb asymmetry.

### 3.2. The Combination of CIMT and EA Does Not Effectively Treat Brain Atrophy

**Figure 3:**
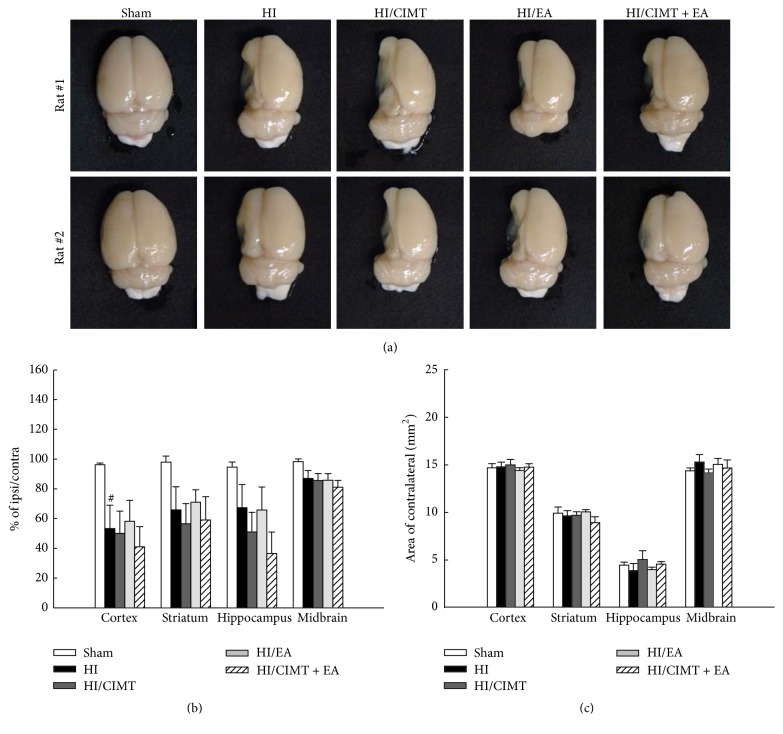
*The combination of CIMT and EA was not effective for reversal of brain atrophy*. (a) Gross photos of the damaged whole brains. (b, c) Brain atrophy was confirmed using Nissl staining and the areas of the cortex, striatum, hippocampus, and midbrain were measured. *N* = 6-7 (per group), ^#^ *P* < 0.05 versus sham group (unpaired *t*-test).

We investigated the ability of combined CIMT and EA to reduce cerebral atrophy following HI injury (Figure 3). In the HI group, all investigated regions of the brain had atrophied and were found to be damaged (Figure 3(a)). Brain atrophy in the ipsilateral regions was not changed in any of the treatment groups (CIMT alone, EA alone, or a combination of the two; Figure 3(b)). We also measured the areas of several contralateral regions of the brain, including the cortex, striatum, hippocampus, and midbrain and found no differences among the groups in these regions (Figure 3(c)).

### 3.3. Combination of CIMT and EA Inversely Regulates the Number of Neurons and Astrocytes in the Cortex

**Figure 4:**
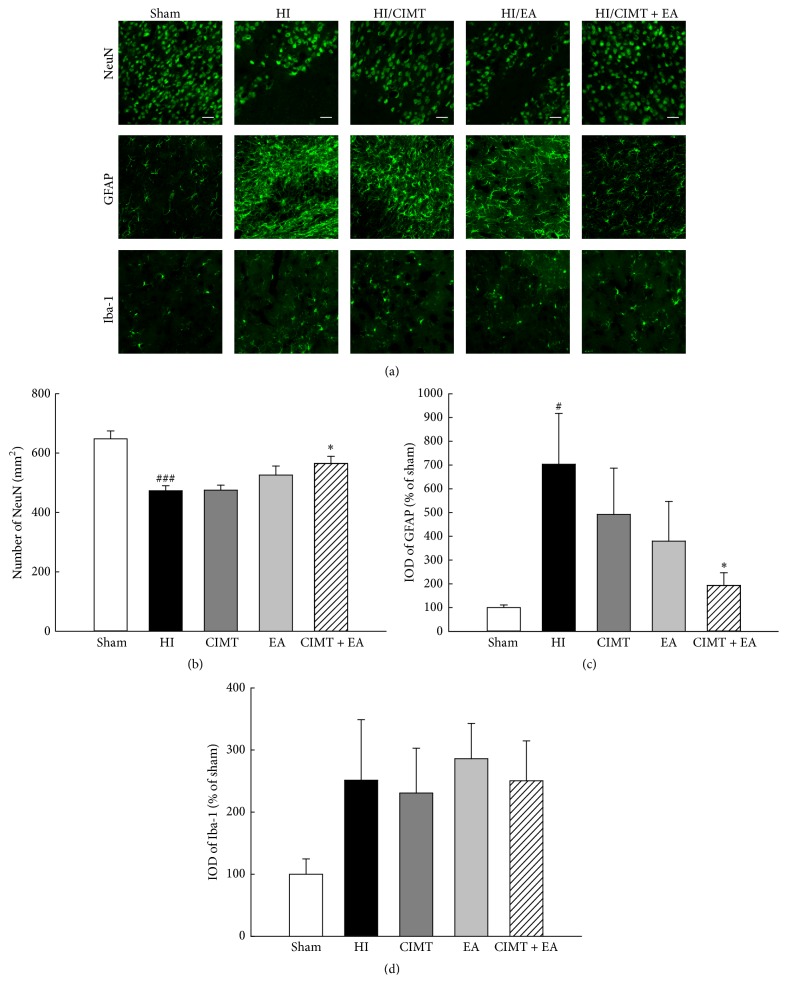
*The numbers of neurons and astrocytes were inversely regulated by combined CIMT and EA*. (a) Immunofluorescence staining for NeuN (neuron marker), GFAP (astrocyte marker), and Iba-1 (labels microglia) in the cortex. Magnification = ×200. Scale bar = 20 *μ*m. (b, c, d) Graphs show number or integrated optical density (IOD) of NeuN, GFAP, and Iba-1 (*N* = 6). ^#^ *P* < 0.05 and ^###^ *P* < 0.001 versus the sham group (unpaired *t*-test). ^*∗*^ *P* < 0.05 versus the HI group (one-way repeated measures ANOVA).

**Figure 5:**
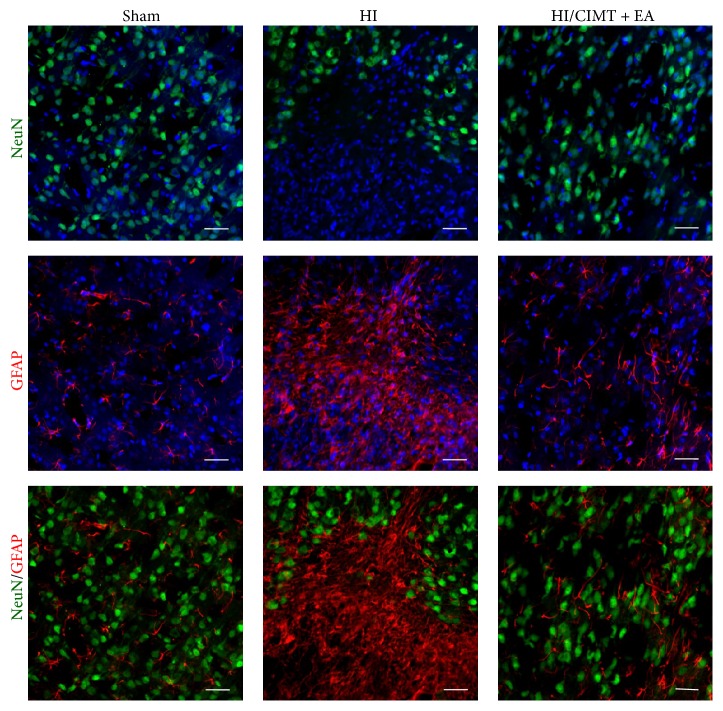
*Immunofluorescence staining for NeuN and GFAP in the cortex*. Double-labeling of NeuN (green) and GFAP (red) shows exclusive expression patterns. Magnification = ×200. Scale bar = 20 *μ*m.

As forelimb asymmetry was improved by combination of CIMT with EA (Figure 2(b)), we investigated the effect of combination treatment on neuron, astrocyte, and microglia in the motor and sensory cortex (Figure 4). NeuN (a neuronal marker) was significantly decreased in the HI group versus the sham group. The number of NeuN-expressing cells was not significantly higher in the HI/CIMT and HI/EA groups versus the HI group, but NeuN was significantly increased in the group that received a combined treatment of CIMT and EA (Figures 4(a) and 4(b)). Further, the expression of GFAP, a marker for astrocytes, was remarkably higher in the HI group versus the sham group and was significantly lower in the HI/CIMT + EA group versus the HI group (Figures 4(a) and 4(c)). Iba-1, a microglial marker, was slightly higher in the HI group versus the sham group, but no significant change was observed in the treatment groups versus the HI group (Figures 4(a) and 4(d)).  Figure 5 shows the distributions of NeuN and GFAP and shows the opposing expression patterns between these markers. That is, NeuN was almost nonexistent in the GFAP positive areas.

### 3.4. Cleaved Caspase-3 Levels Decrease following Combined CIMT and EA

**Figure 6:**
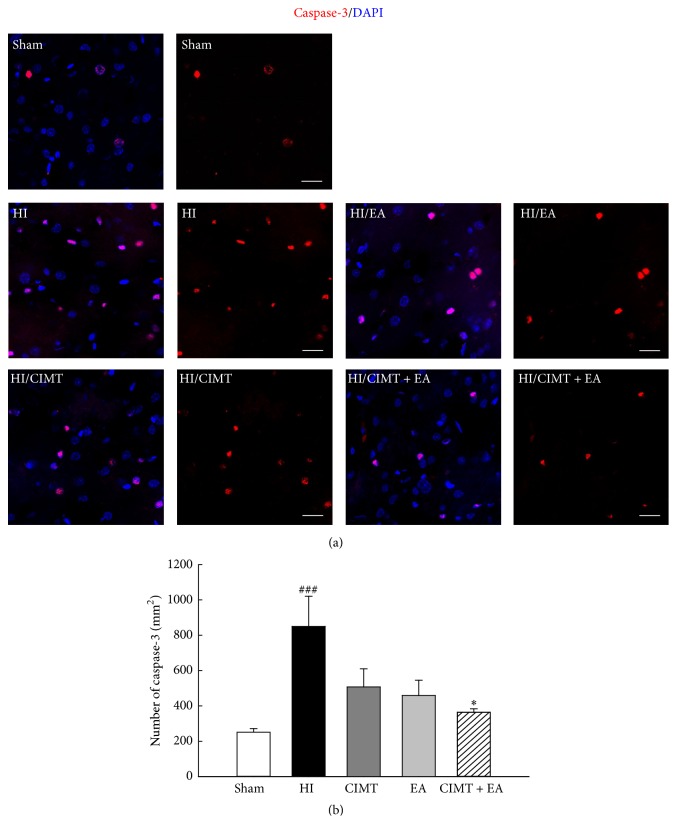
*Cleaved caspase-3 levels were lower in the group that received combined CIMT and EA after HI injury*. (a) Representative confocal images of cleaved caspase-3 (red) in the cortex. Magnification = ×400. Scale bar = 20 *μ*m. (b) Quantification of cleaved caspase-3 levels in the cortex (*N* = 6). ^###^ *P* < 0.001 versus the sham group (unpaired *t*-test). ^*∗*^ *P* < 0.05 versus the HI group (one-way repeated measures ANOVA).

Cleaved caspase-3 levels are known to increase in the brain after neonatal HI injury [31–33]; therefore, we investigated whether cleaved caspase-3 levels were altered by CIMT or EA in our neonatal HI model. Cleaved caspase-3 levels were significantly higher in the HI injury group compared with the sham group (Figure 6), and the levels were smaller in both the CIMT and the EA groups compared with the HI group, although these differences were not significant. Further, cleaved caspase-3 levels were significantly smaller in the group that received a combination of CIMT and EA compared to the HI group (Figure 6). This suggests that a combination of CIMT and EA suppresses caspase-3 and neural cell death in the cortical area after HI injury.

## 4. Discussion

In this study, we investigated the efficacy of combined CIMT and EA by analyzing body weight, motor function, volumetric changes, and histological markers in a rat model of neonatal HI. Our results show that asymmetric locomotor activity was restored through a combination of CIMT and EA. In addition, we observed increased neurons, decreased astrocytes, and reduced cleaved caspase-3 in the cortical area of the HI/CIMT + EA group.

HI brain injury is associated with approximately 25% of neonatal deaths globally [34, 35]. In cases of survival, HI injury causes severe, long-term neurological disorders in infants and children, including cerebral palsy and motor and cognitive deficits [3, 4]. Oxidative stress might be an important contributing factor in perinatal HI brain injury [4], causing an inflammatory response, apoptosis, and necrosis in the neural environment. Therefore, it is crucial to develop an effective treatment to overcome neonatal HI defects.

We found a significant improvement in functional outcomes in the combination CIMT and EA group (Figure 2). CIMT forcibly increased functional use of the damaged arm after HI injury and after stroke [17, 36]. The effects of this therapy can change depending on the intensity of exercise, stress, and motivation [8, 17]. We previously developed a modified CIMT method that was shown to be more effective using repeated exercise during a short treatment time [25]. However, CIMT alone was only minimally effective for functional recovery in a rat HI model [25]. Similar to our results, several reports have shown that CIMT alone does not enhance recovery in an ischemic stroke model [37, 38], although the application of both CIMT and growth factors, such as brain-derived neurotrophic factor (BDNF) or granulocyte-colony stimulating factor, has been shown to improve outcomes after experimental stroke [37, 38]. Further, when CIMT was combined with an enriched environment, neurogenesis was enhanced and functional outcomes were improved [39].

In the current study, we examined the combined effect of CIMT and EA in an experimental model of neonatal HI. EA has been known to stimulate neurotrophic factors in neurological diseases, including stroke [19]. EA improves functional outcomes by increasing the expression of BDNF, glial cell line-derived neurotrophic factor (GDNF), and insulin-like growth factor-1 [22, 40, 41]. It was also reported that EA stimulation improved neuromotor functional recovery and cognitive deficits after ischemic stroke in mice [42]. EA treatment is known to be effective in brain chemistry alteration through the secretion of neurotransmitters and neurohormones and the regulation of blood flow and body temperature around the acupoints [43]. In particular, the acupuncture point of Baihui (GV20) is located in the governor vessels [44] and is mainly used for the treatment of headache, stroke, dizziness, and anxiety. It is also used in ordinary psychological problems because this point shows sedative and harmonizing effects [44]. Zusanli (ST36) is an acupoint located in the stomach median [45, 46]. In the papers by Fang et al. [47], Zusanli (ST36) stimulates the nucleus of the solitary tract. In addition, Zusanli (ST36) affects brain activity and improves gastric mobility, gastric secretion, and gastric electrical activity [45, 46]. The results of the Zusanli studies in stroke are mostly involved in stimulating the cell survival mediators, inhibition of immune cell activity, and apoptosis in the infarction area, rather than activating specific nerves [48, 49]. Therefore, we assumed that acupoints of Baihui and Zusanli activate the nervous system by inducing the secretion of neurotransmitters or neuromodulators from the brain or spinal cord rather than activating specific nerves.

Cell death in response to neonatal HI occurs via distinct types of apoptosis, necrosis, autophagy, and chronic immune response. If this environment is not corrected within a limited time, necrosis and cell death result in nerve damage, functional damage, and death [50]. Upregulated GFAP expression occurs in the damaged area after neonatal HI and stroke [51]. GFAP has been considered a “pan-astrocytic” marker, and an increase in GFAP expression is thus an indicator of an increase in reactive astrocytes [51]. Reactive astrocytes occur in the vicinity of microglia and immune cells, resulting in cytotoxic inflammation [52]. Reactive astrocytes can lead to neuronal cell death by inducing neurotoxicity and permanent damage due to glial scar formation [52]. We observed that the combination of CIMT and EA decreased GFAP positive astrocytes and increased neurons in the cortex (Figures 4 and 5). In addition, the combination of CIMT and EA decreased cleaved caspase-3 levels in the cortex (Figure 6). It has been reported that EA inhibits cerebral ischemic damage by inhibiting reactive gliosis [53] and that CIMT reduces postischemic astrogliosis [38]. Therefore, we speculate that combined CIMT and EA suppress reactive astrocytes and reduce cell death after HI injury. Further experiments should be conducted to clarify the mechanisms by which a combination of CIMT and EA decreases neurotoxicity in a neonatal HI model.

## 5. Conclusion

Our findings show the synergistic effect of combining CIMT with EA compared with applying either of these treatments alone. We suggest that an appropriate combination of various rehabilitation therapies, such as CIMT and EA, is more effective to obtain functional recovery in neonatal HI.

## Supplementary Materials

Supplementary Materials Supplementary Figure S1. Dashed red square indicates the photographed area.

## References

[1] Perlman J. M. (1997). Intrapartum hypoxic-ischemic cerebral injury and cerebral palsy: Medicolegal issues. *Pediatrics*.

[2] Volpe J. J. (2012). Neonatal encephalopathy: an inadequate term for hypoxic-ischemic encephalopathy. *Annals of Neurology*.

[3] Fineschi V., Viola R. V., La Russa R., Santurro A., Frati P. (2017). A Controversial Medicolegal Issue: Timing the Onset of Perinatal Hypoxic-Ischemic Brain Injury. *Mediators of Inflammation*.

[4] Arteaga O., Álvarez A., Revuelta M., Santaolalla F., Urtasun A., Hilario E. (2017). Role of antioxidants in neonatal hypoxic–ischemic brain injury: New therapeutic approaches. *International Journal of Molecular Sciences*.

[5] Graham E. M., Ruis K. A., Hartman A. L., Northington F. J., Fox H. E. (2008). A systematic review of the role of intrapartum hypoxia-ischemia in the causation of neonatal encephalopathy. *American Journal of Obstetrics & Gynecology*.

[6] Dobkin B. H. (2004). Strategies for stroke rehabilitation. *The Lancet Neurology*.

[7] Feys H. M., De Weerdt W. J., Selz B. E. (1998). Effect of a therapeutic intervention for the hemiplegic upper limb in the acute phase after stroke: A single-blind, randomized, controlled multicenter trial. *Stroke*.

[8] Brady K., Garcia T. (2009). Constraint-induced movement therapy (CIMT): Pediatric applications. *Developmental Disabilities Research Reviews*.

[9] Taub E., Uswatte G., Elbert T. (2002). New treatments in neurorehabilitation founded on basic research. *Nature Reviews Neuroscience*.

[10] Cope S. M., Liu X.-C., Verber M. D., Cayo C., Rao S., Tassone J. C. (2010). Upper limb function and brain reorganization after constraint-induced movement therapy in children with hemiplegia. *Developmental Neurorehabilitation*.

[11] Liepert J., Bauder H., Miltner W. H. R., Taub E., Weiller C. (2000). Treatment-induced cortical reorganization after stroke in humans. *Stroke*.

[12] Nudo R. J., Milliken G. W., Jenkins W. M. (1996). Use-dependent alterations of movement representations in primary motor cortex of adult squirrel monkeys. *The Journal of Neuroscience*.

[13] Ishida A., Misumi S., Ueda Y. (2015). Early constraint-induced movement therapy promotes functional recovery and neuronal plasticity in a subcortical hemorrhage model rat. *Behavioural Brain Research*.

[14] Nudo R. J., Wise B. M., SiFuentes F., Milliken G. W. (1996). Neural substrates for the effects of rehabilitative training on motor recovery after ischemic infarct. *Science*.

[15] El-Helow M. R., Zamzam M. L., Fathalla M. M. (2015). Efficacy of modified constraint-induced movement therapy in acute stroke. *European Journal of Physical and Rehabilitation Medicine*.

[16] Psychouli P., Kennedy C. R. (2016). Modified constraint-induced movement therapy as a home-based intervention for children with cerebral palsy. *Pediatric Physical Therapy*.

[17] Eliasson A. C., Krumlinde-Sundholm L., Gordon A. M. (2014). Guidelines for future research in constraint-induced movement therapy for children with unilateral cerebral palsy: An expert consensus. *Developmental Medicine & Child Neurology*.

[18] Yang A., Wu H. M., Tang J., Xu L., Yang M., Liu G. J. (2016). Acupuncture for stroke rehabilitation. *Cochrane Database of Systematic Reviews*.

[19] Shin H. K., Lee S.-W., Choi B. T. (2017). Modulation of neurogenesis via neurotrophic factors in acupuncture treatments for neurological diseases. *Biochemical Pharmacology*.

[20] Wu X.-D., Du L.-N., Wu G.-C., Cao X.-D. (2001). Effects of electroacupuncture on blood-brain barrier after cerebral ischemia-reperfusion in rat. *Acupuncture & Electro-Therapeutics Research*.

[21] Xu H., Zhang Y., Sun H., Chen S., Wang F. (2014). Effects of acupuncture at gv20 and st36 on the expression of matrix metalloproteinase 2, aquaporin 4, and aquaporin 9 in rats subjected to cerebral ischemia/reperfusion injury. *PLoS ONE*.

[22] Zhang Y., Lan R., Wang J. (2015). Acupuncture reduced apoptosis and up-regulated BDNF and GDNF expression in hippocampus following hypoxia-ischemia in neonatal rats. *Journal of Ethnopharmacology*.

[23] Kim H. N., Pak M. E., Shin M. J. (2017). Comparativeanalysis of the beneficial effects of treadmill training and electroacupuncture in a rat model of neonatal hypoxia-ischemia. *International Journal of Molecular Medicine*.

[24] Li H.-B., Wu F., Miao H.-C., Xiong K.-R. (2016). Effects of Polysaccharide of Gastrodia Elata Blume and Electro-Acupuncture on Expressions of Brain-Derived Neurotrophic Factor and Stem Cell Factor Protein in Caudate Putamen of Focal Cerebral Ischemia Rats. *Medical Science Monitor Basic Research*.

[25] Kim H., Kim M. J., Koo Y. S. (2017). Histological and functional assessment of the efficacy of constraint-induced movement therapy in rats following neonatal hypoxic-ischemic brain injury. *Experimental and Therapeutic Medicine*.

[26] Vannucci R. C., Connor J. R., Mauger D. T. (1999). Rat model of perinatal hypoxic-ischemic brain damage. *Journal of Neuroscience Research*.

[27] Vannucci R. C., Rossini A., Grandjean J. (1997). Measuring the Accentuation of the Brain Damage That Arises from Perinatal Cerebral Hypoxia-Ischemia. *Neonatology*.

[28] Kim J. H., Choi K. H., Jang Y. J. (2013). Electroacupuncture preconditioning reduces cerebral ischemic injury via BDNF and SDF-1*α* in mice. *BMC Complementary and Alternative Medicine*.

[29] Yin C. S., Jeong H.-S., Park H.-J. (2008). A proposed transpositional acupoint system in a mouse and rat model. *Research in Veterinary Science*.

[30] Balkaya M., Kröber J. M., Rex A., Endres M. (2013). Assessing post-stroke behavior in mouse models of focal ischemia. *Journal of Cerebral Blood Flow & Metabolism*.

[31] Cheng Y., Deshmukh M., D'Costa A. (1998). Caspase inhibitor affords neuroprotection with delayed administration in a rat model of neonatal hypoxic-ischemic brain injury. *The Journal of Clinical Investigation*.

[32] Blomgren K., Zhu C., Wang X. (2001). Synergistic activation of caspase-3 by m-calpain after neonatal hypoxia-ischemia: A mechanism of "pathological apoptosis"?. *The Journal of Biological Chemistry*.

[33] Northington F. J., Chavez-Valdez R., Martin L. J. (2011). Neuronal cell death in neonatal hypoxia-ischemia. *Annals of Neurology*.

[34] Tagin M., Abdel-Hady H., Rahman S. U., Azzopardi D. V., Gunn A. J. (2015). Neuroprotection for perinatal hypoxic ischemic encephalopathy in low- and middle-income countries. *Journal of Pediatrics*.

[35] Liu L., Oza S., Hogan D. (2015). Global, regional, and national causes of child mortality in 2000–13, with projections to inform post-2015 priorities: an updated systematic analysis. *The Lancet*.

[36] Miltner W. H. R., Bauder H., Sommer M., Dettmers C., Taub E. (1999). Effects of constraint-induced movement therapy on patients with chronic motor deficits after stroke: A replication. *Stroke*.

[37] Diederich K., Quennet V., Bauer H. (2012). Successful regeneration after experimental stroke by granulocyte-colony stimulating factor is not further enhanced by constraint-induced movement therapy either in concurrent or in sequential combination therapy. *Stroke*.

[38] Schäbitz W., Berger C., Kollmar R. (2004). Effect of brain-derived neurotrophic factor treatment and forced arm use on functional motor recovery after small cortical ischemia. *Stroke*.

[39] Rha D.-W., Kang S.-W., Park Y.-G. (2011). Effects of constraint-induced movement therapy on neurogenesis and functional recovery after early hypoxic-ischemic injury in mice. *Developmental Medicine & Child Neurology*.

[40] Gao H., Guo J., Zhao P., Cheng J. (2006). Influences of Electroacupuncture on the Expression of Insulin-like Growth Factor-1 Following Focal Cerebral Ischemia in Monkeys. *Acupuncture & Electro-Therapeutics Research*.

[41] Tao J., Chen B., Gao Y. (2014). Electroacupuncture enhances hippocampal NSCs proliferation in cerebral ischemia-reperfusion injured rats via activation of notch signaling pathway. *International Journal of Neuroscience*.

[42] Kim Y. R., Kim H. N., Ahn S. M., Choi Y. H., Shin H. K., Choi B. T. (2014). Electroacupuncture promotes post-stroke functional recovery via enhancing endogenous neurogenesis in mouse focal cerebral ischemia. *PLoS ONE*.

[43] Tsuchiya M., Sato E. F., Inoue M., Asada A. (2007). Acupuncture enhances generation of nitric oxide and increases local circulation. *Anesthesia & Analgesia*.

[44] Shen E.-Y., Chen F.-J., Chen Y.-Y., Lin M.-F. (2011). Locating the acupoint Baihui (GV20) beneath the cerebral cortex with MRI reconstructed 3D neuroimages. *Evidence-Based Complementary and Alternative Medicine*.

[45] Chen Y., Xu J., Liu S., Hou X. (2013). Electroacupuncture at ST36 increases contraction of the gastric antrum and improves the SCF/c-kit pathway in diabetic rats. *American Journal of Chinese Medicine*.

[46] Yang Q., Xie Y.-D., Zhang M.-X. (2014). Effect of electroacupuncture stimulation at Zusanli acupoint (ST36) on gastric motility: possible through PKC and MAPK signal transduction pathways. *BMC Complementary and Alternative Medicine*.

[47] Fang J.-F., Du J.-Y., Shao X.-M., Fang J.-Q., Liu Z. (2017). Effect of Electroacupuncture on the NTS is modulated primarily by acupuncture point selection and stimulation frequency in normal rats. *BMC Complementary and Alternative Medicine*.

[48] Chen A., Lin Z., Lan L. (2012). Electroacupuncture at the Quchi and Zusanli acupoints exerts neuroprotective role in cerebral ischemia-reperfusion injured rats via activation of the PI3K/Akt pathway. *International Journal of Molecular Medicine*.

[49] Tao J., Zheng Y., Liu W. (2016). Electro-acupuncture at LI11 and ST36 acupoints exerts neuroprotective effects via reactive astrocyte proliferation after ischemia and reperfusion injury in rats. *Brain Research Bulletin*.

[50] Rocha-Ferreira E., Hristova M. (2016). Plasticity in the neonatal brain following hypoxic-ischaemic injury. *Neural Plasticity*.

[51] Choudhury G. R., Ding S. (2016). Reactive astrocytes and therapeutic potential in focal ischemic stroke. *Neurobiology of Disease*.

[52] Liddelow S. A., Barres B. A. (2017). Reactive Astrocytes: Production, Function, and Therapeutic Potential. *Immunity*.

[53] Han X., Huang X., Wang Y., Chen H. (2010). A study of astrocyte activation in the periinfarct region after cerebral ischemia with electroacupuncture. *Brain Injury*.

