# Supplementary figures and images for: Combination of Constraint-Induced Movement Therapy with Electroacupuncture Improves Functional Recovery following Neonatal Hypoxic-Ischemic Brain Injury in Rats
Source: Biomed Res Int. 2018 Feb 7;2018:8638294. doi: 10.1155/2018/8638294 (PMC5820667; doi:10.1155/2018/8638294)

## Slide 1
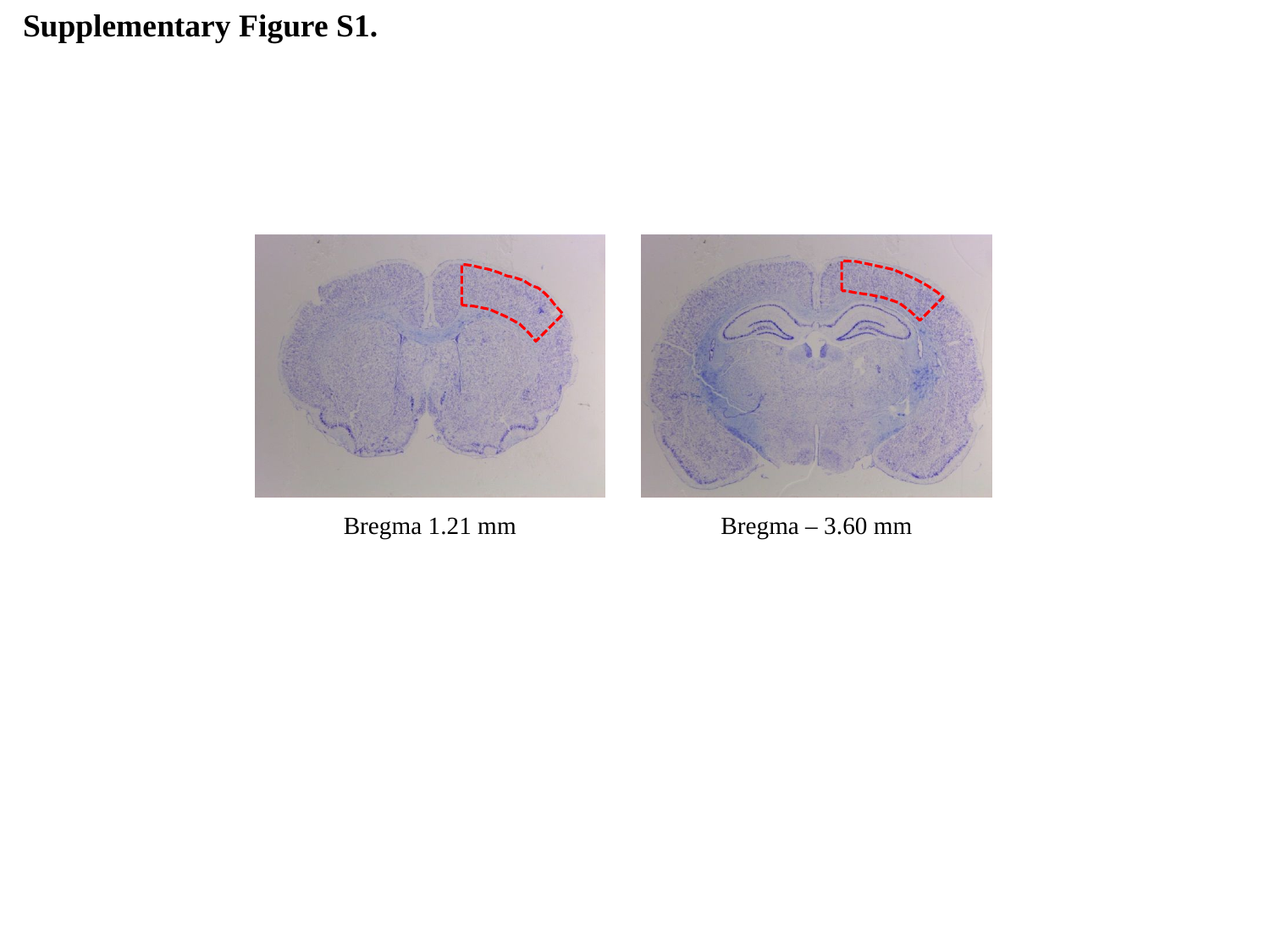

Supplementary Figure S1.
Bregma 1.21 mm
Bregma – 3.60 mm

Supplement: Supplementary Materials — Supplementary Figure S1. Dashed red square indicates the photographed area. [file 8638294.f1.pptx]
